# Deficits in tongue motor control are linked to microstructural brain damage in multiple sclerosis: a pilot study

**DOI:** 10.1186/s12883-015-0451-9

**Published:** 2015-10-08

**Authors:** Florian Holtbernd, Michael Deppe, Rainald Bachmann, Siawoosh Mohammadi, Erich B. Ringelstein, Ralf Reilmann

**Affiliations:** Department of Neurology, RWTH Aachen University, Pauwelsstrasse 30, 52074 Aachen, Germany; Department of Neurology, University of Muenster, Albert-Schweitzer-Campus 1, Building A1, Muenster, Germany; Department of Radiology, Marienhospital Aachen, Zeise 4, 52066 Aachen, Germany; Wellcome Trust Centre for Neuroimaging, UCL Institute of Neurology, University College London, 12 Queen Square, London, WC1N 3BG UK; Department of Systems Neuroscience, University Medical Center Hamburg-Eppendorf, Hamburg, Germany; George-Huntington-Institute, Technology Park Muenster, Johann-Krane-Weg 27, 48149 Muenster, Germany; Department of Neurodegenerative Diseases and Hertie-Institute for Clinical Brain Research, University of Tuebingen, Hoppe-Seyler-Straße 3, 72076 Tuebingen, Germany; Department of Clinical Radiology, University of Muenster, Albert-Schweitzer-Campus 1, Building A1, Muenster, Germany

**Keywords:** Multiple sclerosis, Diffusion tensor imaging, Tongue force, Biomarker, MRI

## Abstract

**Background:**

Deterioration of fine motor control of the tongue is common in Multiple Sclerosis (MS) and has a major impact on quality of life. However, the underlying neuronal substrate is largely unknown. Here, we aimed to explore the association of tongue motor dysfunction in MS patients with overall clinical disability and structural brain damage.

**Methods:**

We employed a force transducer based quantitative-motor system (Q-Motor) to objectively assess tongue function in 33 patients with MS. The variability of tongue force output (TFV) and the mean applied tongue force (TF) were measured during an isometric tongue protrusion task. Twenty-three age and gender matched healthy volunteers served as controls. Correlation analyses of motor performance in MS patients with individual disease burden as expressed by the Expanded Disability Status Scale (EDSS) and with microstructural brain damage as measured by the fractional anisotropy (FA) on Diffusion Tensor Imaging were performed.

**Results:**

MS patients showed significantly increased TFV and decreased TF compared to controls (*p* < 0.02). TFV but not TF was correlated with the EDSS (*p* < 0.04). TFV was inversely correlated with FA in the bilateral posterior limb of the internal capsule expanding to the brain stem (*p* < 0.001), a region critical to tongue function. TF showed a weaker, positive and unilateral correlation with FA in the same region (*p* < 0.001).

**Conclusions:**

Changes in TFV were more robust and correlated better with disease phenotype and FA changes than TF. TFV might serve as an objective and non-invasive outcome measure to augment the quantitative assessment of motor dysfunction in MS.

## Background

Articulatory dysfunction and swallowing disorders are common in multiple sclerosis (MS) and affect up to 60 % of patients [1–3]. The tongue is critically involved in both articulatory function and swallowing. Consequently, it has been suggested that tongue motor function might serve as a surrogate for dysarthria and dysphagia [4, 5]. Indeed, MS patients display reduced tongue muscle strength, premature fatigue, and slowing during repetitive movements even before clinical dysarthria emerges [6]. However, it is not known how these deficits are linked to overall disease burden and the neuropathological substrate of the disease.

Diffusion tensor imaging (DTI) has evolved as a reliable method to quantify the severity of brain tissue damage in MS. Specifically, reductions of the fractional anisotropy (FA) in the cerebral white matter have consistently been reported in MS patients [7]. In this vein, we have found previously that decreased FA in the cerebral white matter adjacent to sensory and visual cortices was linked to increased grip force variability in patients with MS [8].

Here, we utilized a force transducer based experimental setup to objectively quantify deficits in the fine motor control of the tongue in a cohort of MS patients and explored whether these deficits were associated with overall clinical disability and brain microstructural integrity.

## Methods

### Subjects

Thirty-three patients with the diagnosis of MS were recruited through the Department of Neurology at the University Hospital of Muenster. Their mean age was 38.8 years (SD +/−10.5, range 19–61), 23 were females. The mean EDSS was 3.8 (+/−1.9, range 1–7.5). Eighteen patients suffered from relapsing-remitting MS (RRMS), 11 from secondary progressive MS (SPMS) and 4 from primary progressive MS (PPMS). Twenty-three age-matched healthy control subjects (38.4 +/−9.3 years, range 24–55, 16 females) with no former history of neurological or psychiatric disease served as controls. Demographical and clinical data of MS patients and controls are summarized in Table 1. Prior to study participation, informed consent was obtained from each participant in accordance with the Declaration of Helsinki, and the study was approved by the local ethics committee at the University Clinic of Muenster. The majority of MS patients and all control subjects have been part of a previously published study investigating grip force control in MS [8].

**Table 1:** Demographical and clinical data of patients with multiple sclerosis and healthy controls

	Age	Gender	EDSS	Type of MS
MS patients (*n* = 33)	38.8 ± 10.5 (19–61)	23 F/10 M	3.8 ± 1.9 (1–7.5)	18 RRMS
				11 SPMS
				4 PPMS
Healthy controls (*n* = 23)	38.4 ± 9.3 (24–55)	16 F/7 M	-	-

*MS* multiple sclerosis, *EDSS* expanded disability status scale, *RRMS* relapsing remitting multiple sclerosis, *SPMS* secondary progressive multiple sclerosis, *PPMS* primary progressive multiple sclerosis; values are presented as mean ± SD (range)

### Isometric tongue force assessment (Glossomotography)

The experimental setup of the Q-Motor “glossomotography” device (QuantiMedis GmbH, Muenster, Germany) has been described in detail elsewhere [9]. Briefly, the subjects protruded their tongue to establish contact with a circular pre-calibrated force transducer mounted on a glossomotograph. The subjects had clear sight at a monitor positioned 30 cm in front of the apparatus. A horizontal line indicated the target force. Subjects were instructed to match the indicated force level by generating an appropriate isometric tongue protrusion force. Each trial lasted 30 s, a cueing tone signaled start and end of each trial. Five trials each were performed at a target force level of 0.5 [N]. Data was sampled at 400 Hz, stored and analyzed on a laboratory computer system (SC/ZOOM, University of Umea, Sweden). The applied mean tongue protrusion force (TF) and the tongue force variability (TFV; defined as the coefficient of variation: SD(TF)/TF×100[%]) were measured during the static phase from second 10 to 30 of each trial. Values obtained from each subject were averaged across trials and entered into statistical analyses.

### DTI protocol and data processing

28 of the 33 MS patients were available for DTI imaging as described previously [8]. Controls did not undergo MRI. MRI was conducted using a 3 T whole-body scanner (Gyroscan Intera T30, Philips, Netherlands). Data were acquired using a single shot echo planar imaging (EPI) sequence in 72 axial slices (1.8 mm thick, no gap, FOV 230 × 230 mm, acquired matrix 127 × 128, b factors: 0 and 1,000 s/mm^2^ 6 gradient directions, 3 averages). For further processing all EPI images were reconstructed to 2.0 × 2.0 × 2.0 mm^3^. All images were spatially registered by the multicontrast image registration toolbox for optimal spatial pre-processing of DTI data prior to statistical analysis [10] and corrected for eddy currents in all three dimensions using a recently developed technique [11, 12]. After image registration, all DTI images corresponded to Montreal Neurological Institute (MNI) coordinate space. Following registration, data were spatially smoothed (4 mm FHWM).

FA maps for each subject were calculated and voxel based statistics were applied using SPM (http://www.fil.ion.ucl.ac.uk/spm). Correlational analyses of tongue force measures with FA maps were performed on a voxel by voxel basis using the “simple regression” model implemented in SPM. We applied a voxel threshold of *p* < 0.001, cluster level corrected at *p* < 0.05. The cluster extend cutoff was set at 50 voxels. For *post-hoc* volume of interest (VOI) analysis, FA values were extracted from 4 mm spheres centered at the peak voxel of each cluster revealed by the voxel wise searches and correlated with behavioral measures.

### Statistical analysis

Statistical analysis was performed using SPSS®. The Student’s *t*-test was used for intergroup comparisons of behavioral data between MS patients and healthy controls. The Spearman correlation coefficient was applied for correlation of TF and TFV measures with the EDSS. *Post-hoc* regression analyses of regional DTI measures with TF and TFV, respectively, were carried out using the Pearson correlation coefficient. Results were considered as significant at *p* < 0.05.

## Results

### Intergroup comparisons

**Fig. 1:**
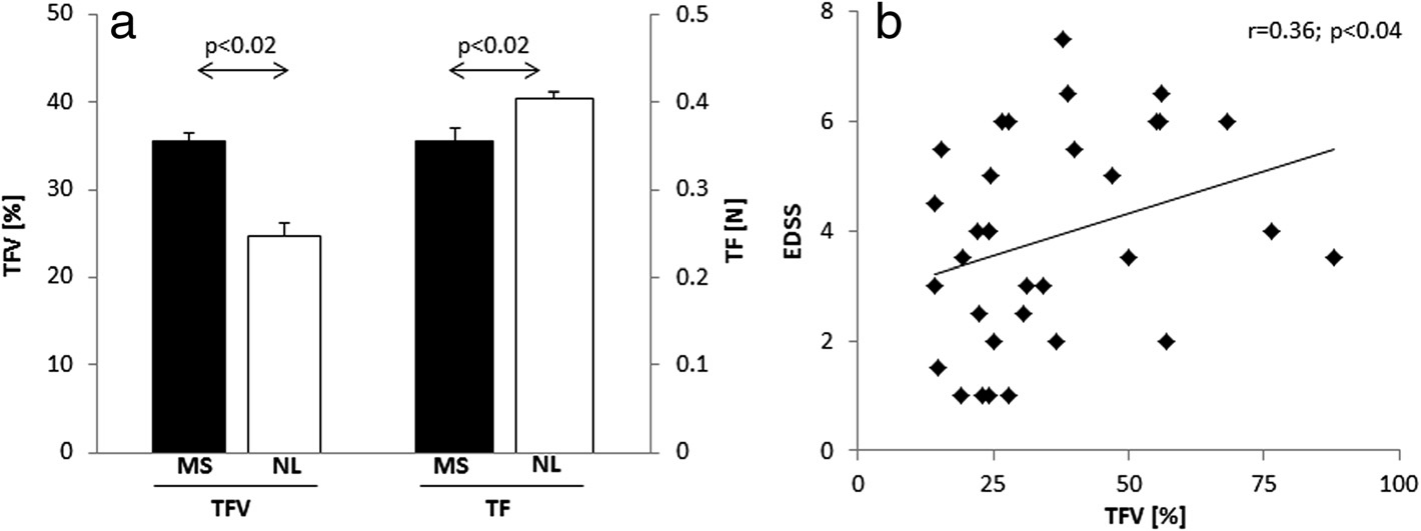
**a** Group comparisons of tongue force variability (TFV) and mean applied tongue force (TF) between patients with Multiple Sclerosis (MS) and normal controls (NL) during an isometric tongue protrusion task. Compared to NL, TFV was significantly increased in MS patients, whereas TF was significantly decreased in the latter group. **b** TFV in MS patients correlated with the overall disease burden as assessed by the Expanded Disability Status Scale (EDSS). Bars express group mean values; the error bars indicate the SEM

TFV was significantly increased in MS patients (35.5+/−18.7 [%] (mean+/−SD)) compared to controls (24.7+/−7.1 [%]; p < 0.02; Fig. 1a*left*). In contrast, TF was significantly decreased in the MS group (0.36+/−0.08 [N] vs. 0.40+/−0.04 [N]; *p* < 0.02; Fig. 1a*right*). Notably, TFV and TF were inversely correlated in patients (r = −0.89, p < 0.001; Pearson correlation coefficient), whereas we did not observe a significant correlation of these measures in the control group (*p* > 0.16).

### Correlation to disease severity

We found a moderate correlation of TFV with the EDSS (*r* = 0.36, *p* < 0.04) (Fig. 1b). TF did not correlate with the EDSS (*p* > 0.10).

### Correlation of behavioral measures with FA

**Fig. 2:**
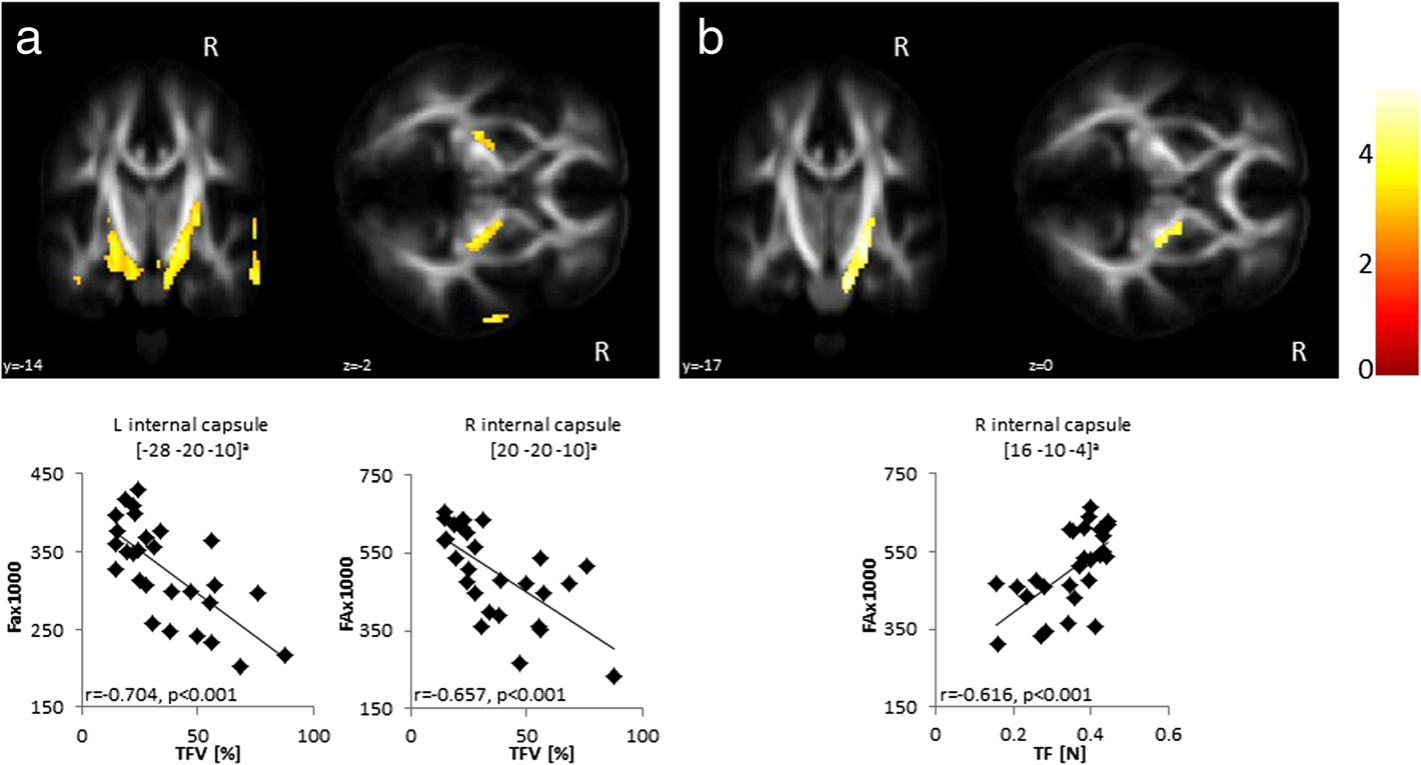
Correlation of tongue force variability (TFV) and mean tongue force (TF) with fractional anisotropy (FA) in 28 patients with Multiple Sclerosis. Results were superimposed on a standard FA template. **a** Symmetric, negative correlations were found between TFV and FA in the posterior limb of the internal capsule expanding to the brain stem. **b** TF exhibited a positive correlation with FA in the right internal capsule only. *Post-hoc* analyses of individual FA values extracted from volumes of interest centered at the peak voxel of each cluster confirmed significant correlations of FA with TFV (**a** *bottom panels*) and TF (**b** *bottom panel*). ^a^Coordinates are displayed in the Montreal Neurological Institute (MNI) standard space; the colored bar indicates the *T*-values; voxel threshold p < 0.005

TFV was inversely correlated with FA in a region encompassing the posterior limb of the internal capsule expanding to the brain stem (Fig. 2a, *top*). Given the inverse relationship of TFV with TF, not surprisingly, we observed a positive, albeit weaker correlation of TF with FA unilaterally in the posterior portion of the right internal capsule (Fig. 2b, *top*). *Post-hoc* VOI analysis confirmed a significant correlation of FA with TFV and TF in the posterior internal capsule (*p* < 0.001; Fig. 2 a/b, *bottom panels*).

## Discussion

We found an increased variability of force output and concurrent reductions of the applied mean force during an isometric tongue protrusion task in MS patients. These findings are in line with previous studies reporting reductions of the voluntary force output and increased force variability in MS patients in a variety of motor tasks [8 ,13, 14]. We have found earlier that the variability of force output showed higher sensitivity than the mean applied force in assessing alterations of motor hand function in MS and other neurological conditions, particularly when deterioration of motor performance was still mild [8, 15, 16]. Along these lines, it is noteworthy that TFV but not TF was associated with overall disability and also exhibited stronger correlations with FA measures. These findings further support the hypothesis of force variability being superior over mean applied force to quantify motor dysfunction and its association to microstructural brain damage in MS [8].

FA measures the degree to which overall diffusion of water molecules can be described as anisotropic. FA ranges from 0–1, where 0 indicates completely isotropic diffusion with no contribution of anisotropic diffusion. FA typically is high (i.e. diffusion is predominantly anisotropic) in brain regions rich in myelinated fibers such as the pyramidal tract or corpus callosum and relatively low (i.e. diffusion is predominantly isotropic) in gray matter areas [17]. While other DTI metrics (e.g. axial and radial diffusivity) have been reported in addition to FA, the latter might be the DTI measure best established and most widely utilized to assess white matter integrity and its association with clinical disability in MS (see e.g.[17, 18] for review). We deliberately chose to measure FA for correlational analyses with behavioral data based on our prior findings. In the latter study, we explored the association of altered hand motor function with cerebral white matter integrity. We found that only FA in the white matter adjacent to the primary sensory and visual cortices, but not other DTI measures, i.e. mean diffusivity, axial diffusivity and radial diffusivity, correlated with quantitative measures of hand motor function, suggesting that FA might be most sensitive to assess white matter damage associated with motor dysfunction.

Supporting these findings, TFV was inversely correlated with FA in the posterior limb of the internal capsule expanding to the brain stem, whereas TF showed a positive correlation with FA in this region. Thus, decreased microstructural white matter integrity was indicative of abnormally high tongue force variability and reduced total force output in the MS patients. The posterior portion of the internal capsule mainly incorporates cortico fugal motor fibers comprising the pyramidal and corticobulbar tracts, and somatosensory fibers. The motor fibers have a somatotopic organization with the tongue being located in the anterior part of the posterior limb [19, 20]. Hence, structural damage of this structure, particularly its anterior portion, is likely to affect tongue function by disrupting motor output from the motor cortex to the tongue [21].

Dysphagia and dysarthria are common in MS. A recent meta-analysis conducted by Guan et al. showed that at least one third of MS patients suffer from dysphagia [22]. Similar estimates have been suggested for dysarthria in MS patients [1]. Dysphagia can emerge in very early disease stages in ambulatory patients, and constitutes a major hazard in severely affected patients. Several studies established a close relationship between overall clinical disability and the presence of dysphagia, suggesting that dysphagia is most pronounced in clinically severely affected patients with an EDSS of 6.5 or higher [2, 3]. Whereas dysphagia is associated with overall clinical disability, it seems that the MS subtype is not a strong predictor of dysphagia [23]. However, reports are equivocal and some authors suggest that patients with progressive MS forms (SPSS and PPMS) are more likely to develop dysphagia compared to patients with RRMS [24]. Similarly, the severity of dysarthria has been found to correlate well with overall disease burden [25], even though subtle signs of dysarthria have been revealed in MS patients without manifest speech disorder [1]. In line with these findings, we found that MS patients with high EDSS scores exhibited pronounced variability of tongue force output.

We acknowledge several limitations of this study. Because of the limited sample size we could not explore potential differences in FA and its correlation with tongue function for the MS subtypes in the current set of data. Moreover, the correlations observed do not necessarily imply a causal relationship of structure and function and the specificity of the findings reported remains unclear because healthy controls did not undergo MRI. That said, the strong and symmetric correlations of TFV with FA in a region crucial to tongue motor function are intriguing and indicate a link of increased TFV to the neuropathological substrate of the disease. Previous reports suggest that both dysarthria and dysphagia are associated with overall disease burden rather than alterations of single functional systems or subtype of MS [22, 26]. That being said, for future studies, it might be worthwhile to additionally apply clinical rating scales specifically assessing dysphagia and dysarthria. Finally, we note that FA is very sensitive to microstructural brain changes *per se* [11, 27], but is limited in characterizing the pathological processes underpinning these structural alterations. For example, FA changes in afferent pathways can be caused by both potentially reversible demyelination and irreversible axonal degeneration in patients with MS [28]. A multi-modal imaging approach including myelin sensitive MRI techniques might help to better understand the pathological processes underlying microstructural tissue damage [29, 30].

## Conclusions

Our findings suggest that the quantitative motor (Q-Motor) measurement of tongue function might proof useful as a non-invasive and cost efficient method to assess motor dysfunction in MS in addition to categorical scales such as the EDSS. Avoidance of inter- and intra-rater variability and lack of site-effects due to the pre-calibration of sensors applied may increase sensitivity and reliability, e.g., in small proof-of-concept studies. In line with prior studies [8, 9] TFV was a more sensitive surrogate of motor dysfunction and more robustly linked to overall disease burden and microstructural brain integrity than TF. Prospective studies are warranted to prove the utility of TFV to assess longitudinal changes of motor phenotype and cerebral microstructure in patients with MS.
